# Evaluation of Over-The-Row Harvester Damage in a Super-High-Density Olive Orchard Using On-Board Sensing Techniques

**DOI:** 10.3390/s18041242

**Published:** 2018-04-17

**Authors:** Manuel Pérez-Ruiz, Pilar Rallo, M. Rocío Jiménez, Miguel Garrido-Izard, M. Paz Suárez, Laura Casanova, Constantino Valero, Jorge Martínez-Guanter, Ana Morales-Sillero

**Affiliations:** 1 Dpto. Ingeniería Aeroespacial y Mecánica de Fluidos, Área de Ingeniería Agroforestal, Escuela Técnica Superior de Ingeniería Agronómica (ETSIA), Universidad de Sevilla, 41013 Sevilla, Spain; martinezj@us.es; 2 Dpto. Ciencias Agroforestales, Escuela Técnica Superior de Ingeniería Agronómica (ETSIA), Universidad de Sevilla, 41013 Sevilla, Spain; prallo@us.es (P.R.); rjg@us.es (M.R.J.); maripaz@us.es (M.P.S.); laucaler@us.es (L.C.); amorales@us.es (A.M.-S.); 3 Laboratorio de Propiedades Físicas (LPF_TRAGRALIA), Universidad Politécnica de Madrid, 28040 Madrid, Spain; miguel.garrido.izard@upm.es (M.G.-I.); constantino.valero@upm.es (C.V.)

**Keywords:** *Olea europaea*, laser scanning, monitoring, canopy volume, fruit damage, olive harvester

## Abstract

New super-high-density (SHD) olive orchards designed for mechanical harvesting using over-the-row harvesters are becoming increasingly common around the world. Some studies regarding olive SHD harvesting have focused on the effective removal of the olive fruits; however, the energy applied to the canopy by the harvesting machine that can result in fruit damage, structural damage or extra stress on the trees has been little studied. Using conventional analyses, this study investigates the effects of different nominal speeds and beating frequencies on the removal efficiency and the potential for fruit damage, and it uses remote sensing to determine changes in the plant structures of two varieties of olive trees (‘Manzanilla Cacereña’ and ‘Manzanilla de Sevilla’) planted in SHD orchards harvested by an over-the-row harvester. ‘Manzanilla de Sevilla’ fruit was the least tolerant to damage, and for this variety, harvesting at the highest nominal speed led to the greatest percentage of fruits with cuts. Different vibration patterns were applied to the olive trees and were evaluated using triaxial accelerometers. The use of two light detection and ranging (LiDAR) sensing devices allowed us to evaluate structural changes in the studied olive trees. Before- and after-harvest measurements revealed significant differences in the LiDAR data analysis, particularly at the highest nominal speed. The results of this work show that the operating conditions of the harvester are key to minimising fruit damage and that a rapid estimate of the damage produced by an over-the-row harvester with contactless sensing could provide useful information for automatically adjusting the machine parameters in individual olive groves in the future.

## 1. Introduction

The olive tree crop occupies 11.5 × 10^6^ ha worldwide and is found from the Mediterranean Basin, where it was first introduced into cultivation more than 3000 years ago, to countries as far away as Australia, the United States, Chile and Argentina [1]. The increase in worldwide olive oil consumption continues to encourage the expansion of this crop. Traditional olive orchards, with an average density of fewer than 100 trees ha^−1^, were designed when manual work was the only known system used to perform the necessary crop operations. Intensification of olive orchards started more than 40 years ago by increasing the number of trees per hectare and led to the mechanisation of harvesting as a method of reducing cultivation costs. Trunk shakers were the first machines used for harvesting traditional olive groves. They are still being used, despite problems associated with separation of the cambium (stripping bark) at the attachment point of the clamp to the trunk, and/or dislodging of roots, both of which may reduce tree longevity [2]. Today, olive trees are cultivated in high-density hedgerows, at 250–400 trees ha^−1^, or super-high density hedgerows (SHD), at 1500–2000 trees ha^−1^ [3]. Both planting densities are well suited for using straddle harvesters, which work over the row by continuously beating the tree canopy, where most fruits are concentrated [4].

Mechanical harvesting requires a compromise between the efficient removal of fruits and the damage to the canopy and the fruits caused by this harvesting. This balance is necessary to ensure the longevity of the orchards, particularly those for which the final products are extra virgin oil and/or high-quality table olives. Previous research on SHD hedgerows show that the efficiency of fruit removal by straddle harvesters is close to 100%, and the total time to harvest rarely exceeds 3–4 h per hectare [3,4,5], although these studies lack details about how much fruit is collected by the harvester and how much falls to the ground. Concerning fruit damage, bruised and cut fruits have been reported at an early stage of fruit maturation when removed using a straddle harvester [5]. These types of damage have also been described in fruits harvested with trunk shakers and are primarily related to the impacts suffered by fruit falling through the canopy into the harvester, although handling and transportation to industrial sites may also increase the severity of damage. Bruising is characterised by dark spots that develop on the skin, deteriorating the fruit’s external appearance, which can be characterised automatically by image analysis [6]. However, ruptured cells and a loss of cell wall thickness have also been reported [7,8]. Losses in the oil quality, such as increased free acidity and decreases in natural antioxidants and flavour components, have also been described in different varieties of fruits growing under SHD conditions that were harvested at later stages of maturation [9,10]. In terms of canopy damage, different tree organs (e.g., shoots, trunk, leaves, etc.) may be severely damaged by the harvester, which can also increase the susceptibility of the tree to diseases. The use of Cu products after harvest is usually recommended to prevent infections. The extent to which the working conditions of the harvester cause damage to the tree canopy is not well known. In general, the fruit detachment and tree damage depend on the amount of shaking/beating energy that is transferred to the tree limbs by the harvester. More fruit is removed as more energy is transferred; however, more energy may also result in more tree damage [11]. The force needed for fruit removal depends on the cultivar and the stage of maturity [12], but it is fairly high (2–12 N·g^−1^) [13], and this force increases the risk of trunk, branch, and fruit damage [3].

Most evaluations and comparative studies on olive harvesters include visual evaluation of stripped bark or broken branches [14] and a quantification of the damage inflicted on the trees, with the consequent consumption of time and effort. The direct measurement of the structural properties of the canopy is time-consuming and labour-intensive, requiring destructive leaf sampling. Therefore, a more efficient contact-free automated system that can quickly quantify harvester-induced damage to the tree is necessary. The purpose of this system is to configure the machinery automatically and in real time to allow the harvest to proceed with minimal damage to the crop. Indirect approaches based on measurements of radiation transmission through the canopy [15,16], image analysis, ultrasonic sensors, and LiDAR sensors [17] are suitable for rapidly obtaining a large amount of data.

Ground-based LiDAR scanners mounted on an off-road vehicle have also been used in canopy structural studies [18]. Geometric definition and measurements using LIDAR have been performed in different orchards, such as vineyards, citrus trees [19,20], fruit orchards [21] or tomato crops [22]. As a result of these characterisations, in most cases, certain tasks, such as accurate pruning or the variable application of agrochemicals, are performed according to the crown volume and tree shape [23]. Many different applications of ground-based LiDAR systems can be found in the agricultural environment, including navigation/auto guidance [24], weed control [25], safety [26], crop characterisation [27], intra-row plant spacing measurement [28], 3D plant reconstruction [29] and tree crop characterisation [30,31].

In the present work, non-invasive methods using LiDAR sensors for monitoring olive harvester-induced canopy changes were used. Furthermore, the study also investigated the effect of the harvester travel speed at certain beating frequencies on olive fruits as well as the fruit removal efficiency and fruit harvester-induced damage that occur in two different varieties of SHD olive orchards. This approach had the following specific objectives:

- to develop a novel platform combining two LiDAR sensor scans in different orientations to select the most reliable one for measuring the olive tree crown volume,
- to evaluate the proposed methodology for identifying structural changes related to the tree damage caused by harvesting, and
- to relate the tree structure changes to fruit removal and the possible damage it caused.

## 2. Materials and Methods

### 2.1. Field Site and Experimental Design

Field trials were performed during the harvesting season in 2016 in a commercial olive orchard located in Campo Maior (Portugal) (latitude: 38°55′55.1″ N; longitude: 7°02′36.8″ W). Nine-year-old trees were planted at 3.75 m intervals between rows and 1.35 m intervals within rows (1975 trees ha^−1^) in a north-south orientation, with drip irrigation. The trees were trained to a central leader system. The ‘Manzanilla de Sevilla’ hedgerow dimensions were 2.5 ± 0.3 m in height and 1.5 ± 0.2 m in width. The ‘Manzanilla Cacereña’ hedgerows had similar heights and widths at 2.4 ± 0.2 m and 1.4 ± 0.3 m, respectively. Therefore, these dimensions were suitable for mechanical harvesting by a straddle harvester. During the previous winter, mechanical pruning was performed between two rows of trees using a tractor equipped with a disc-pruning machine. Thick branches and stumps were later removed with a chainsaw or pruning shears.

**Figure 1:**
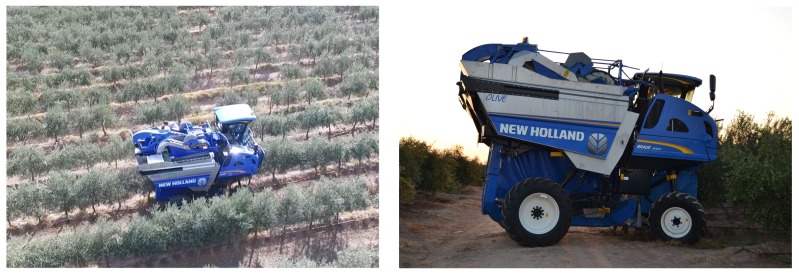
Straddle harvester used in the trial.

The harvesting operation was performed using a straddle harvester (New Holland Braud 9090X Olive, CNH Global, Zedelgem, Belgium) (Figure 1). This machine is characterised by a beater with bow-shaped poles, a conveyor belt transport system with buckets, and a mixed pneumatic and mechanical cleaning apparatus comprising four extractors and a stalk remover. Two of the extractors were positioned beneath the beater, and two were above the stalk remover. The ‘Manzanilla Cacereña’ variety was harvested on September 26th and the ‘Manzanilla de Sevilla’ variety was harvested on the 30th of the same month, after the pigmentation of the skin became green-yellowish.

Three harvesting treatments (i.e., 3 km/h and 470 Hz, 2 km/h and 470 Hz and 2 km/h and 430 Hz for the nominal speed and beating frequency) were established. Beating frequencies lower than 430 Hz were discarded because the fruit removal was negatively affected. The experimental design was based on three random rows of trees (approximately 90 trees/row) per variety and per harvesting treatment.

### 2.2. Harvesting Efficiency

The harvesting efficiency was evaluated by finding the average time of harvest (h·ha^−1^), fruit removal (%), and the percentage (%) of fruit that was dropped on the ground. The time of harvest was determined by measuring the time needed by the harvester to harvest each row of trees. The fruit removal was determined from the total production and the weight of fruit that remained after the harvest, which was estimated for 10 random trees per row. The percentage of fruit that dropped on the ground allowed for the determination of the proportion of fruits intercepted by the harvester, and it was estimated for each row according to the weights of the fruits that fell in an area measuring 2.5 × 2.5 m.

### 2.3. Olive Fruit Damage Caused by Mechanical Harvesting

Immediately after harvesting, three-kilogram samples for each experimental unit were randomly selected from the total amount of harvested fruit. The mean fruit weight (g) was measured using 0.5 kg of fruit. The firmness and colour were measured in the equatorial zone of 30 fruits (two measurements per fruit). The fruit firmness (N cm^−2^) was measured with a Zwick 3300 hand force meter (Zwick GmbH & Co., Ulm, Germany). The fruit colour parameters were determined with a Minolta CM-700d (Konica Minolta Inc., Tokyo, Japan) spectrophotometer, and the colour index (CI) was calculated using the following equation [32]:

*CI* = L* × (b* − a*) × 10^−2^ (1)

where L* denotes the lightness, a* denotes the colour axis from green to red, and b* denotes the colour axis from blue to yellow. The International Commission on Illumination colour notation system was used to determine these parameters. The fruit damage was estimated in a sub-sample of 100 fruits by evaluating the bruising incidence (BI) and the proportion of fruits with cuts two hours after the harvest. For BI, the fruits were classified according to the bruising severity on the skin into the following categories: non-bruised, low damage (<25% of the skin surface affected) and severe damage (25% to 100% of the skin affected). Number of fruits within each category were counted (*N*0, *NL* and *NS*, respectively), and the following equation was applied:

(2) $$BI=\frac{N0\;x\;0+NL\;x\;1+NS\;x\;2}{N0+NL+NS}$$

where *N*0, *NL* and *NS* are the numbers of non-bruised, low-damage and severe damaged bruised fruits, respectively.

### 2.4. Statistical Analysis

An analysis of variance was performed to determine the effects of the harvest treatments on the different varieties. The data were previously transformed using the arcsine of the square root or Box-Cox power transformations [33] when necessary to achieve normality and homogenise the variance. The mean bruising incidence values were calculated, and contingency tables were constructed. The Statgraphics Plus 5.1 (Statpoint Technologies, The Plains, Virginia) software package was used.

### 2.5. Structural Olive Tree Damage Caused by Mechanical Harvesting

To calculate the structural damage caused to the olive trees by the straddle harvester, a novel methodology was designed based on the following factors: (i) the use of two LiDAR sensors in different orientations to compute the density of the point cloud generated before and after the harvesting machine operated on each monitored tree-row transect, (ii) using these point clouds to calculate and compare the volume of biomass occupied by the trees before and after mechanised harvesting, and (iii) directly measuring the vibration transmitted to the tree at different speeds during the harvest.

Trials were established so that the incidence was measured in the plots corresponding to the two olive varieties at different combinations of forward speed and stroke frequency for the machine.

The tree row transects chosen for scanning and monitoring had approximate lengths of 15 m. Two box-shaped elements were used as fixed references to delimit the row transect, as described in [27], to ensure that its position before and after harvest did not vary.

For the odometry system, an incremental optical encoder (model 63R256, Greyhill Inc., Chicago, IL, USA) was directly connected to an unpowered ground wheel to provide a resolution of 3 mm per pulse in the direction of travel. The cumulative odometry pulse count was collected using a low-cost open-hardware Arduino Leonardo microcontroller (Arduino Project, Ivrea, Italy) and then used to determine the instantaneous location of the data along the row, thus serving as a local reference system.

#### 2.5.1. LiDAR Data Acquisition in Field Tests

**Figure 2:**
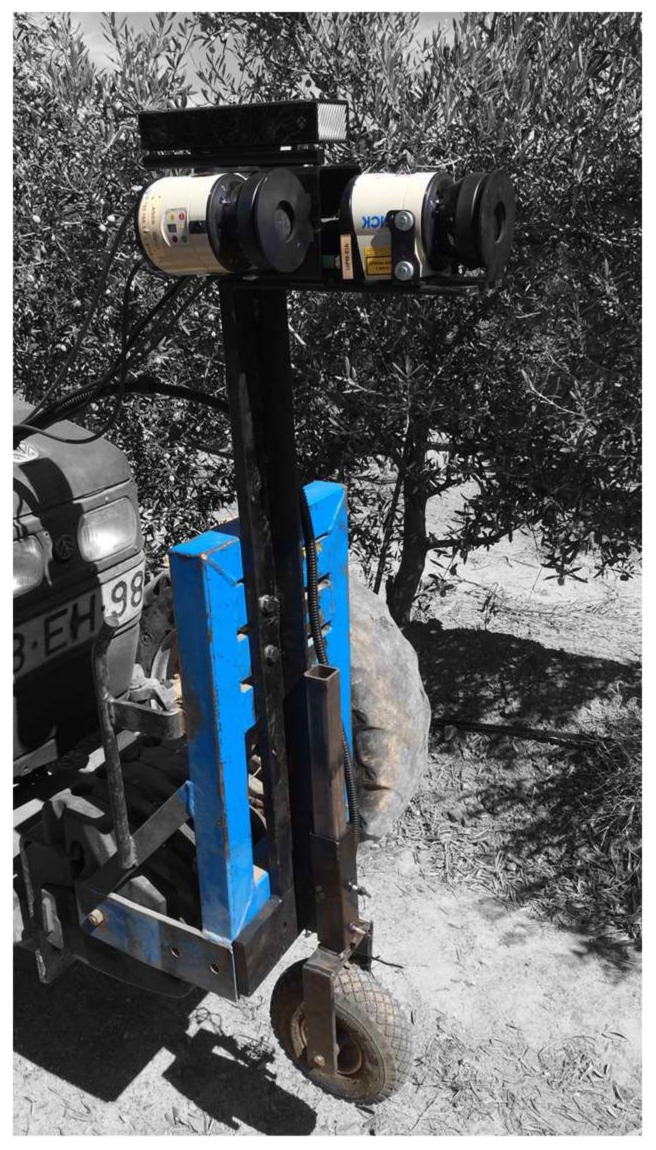
Two LiDAR sensors were mounted on the front of the tractor in different orientations to scan the olive tree structure.

Two LiDAR LMS111 (SICK AG, Waldkirch, Germany) sensors were installed with different orientations on a dedicated platform attached to the front of a tractor at a height of 1.55 m above ground level, vertically (facing upwards) and laterally (facing sideways) (Figure 2). The LiDAR technical features are summarised in Table 1. The purpose of this scan configuration was to compare the data collected by each sensor. Point clouds generated by the vertically installed LiDAR sensors were filtered to obtain the same tree’s lateral projection with both sensors. The LiDAR sensors were interfaced with a computer through an RJ45 Ethernet port for data transfer. The resolutions of both LiDAR sensors in the direction of travel were greatly affected by the tractor speed. Maintaining a constant speed was a key factor for obtaining accurate measurements. LiDAR data were recorded using a self-developed Lab View program (National Instruments Co., Austin, TX, USA). Both LiDAR sensors collected distance values in 271 directions (corresponding to a scanning field of view of 270°) every 20 ms (50 Hz) at half-degree angular steps to obtain the lateral projection of the tree with both sensors.

#### 2.5.2. LiDAR Data-Processing Methodology

This section describes the methodology proposed to obtain aerial point clouds from olive tree rows using the wheel encoder sensor and the damage estimation, and the methodology was based on the volume difference in the tree biomasses. For this purpose, it was necessary to represent the point cloud, followed by transforming and aligning the resulting scans, to finally calculate and compare the volumes of each point cloud.

##### Point Cloud Representation

For the offline representation of the data collected in the field, a self-developed program (using the Python programming language) was used, combining the NumPy and Point Cloud Library analysis libraries. For this representation, to adjust the point clouds to the Cartesian coordinate system that corresponded to the actual orientation of the LiDAR, the transformations of the angles of the roll, pitch and yaw angles of the sensors (as shown in Table 2) were integrated into the program.

Once transformed, the x-translation was applied to coordinates obtained for the actual LiDAR orientation. The encoder values recorded at each scan time were used to update the point cloud x-coordinate related to the tractor advance.

Using this approach, data corresponding to tests on the left and right side of each row were represented before and after the harvest (BH and AH), with the two LiDAR sensors treated separately (facing sideways and facing upwards), resulting in 8 independent point clouds for each transect on the tests.

##### Point Cloud Alignment and Filtering

**Figure 3:**
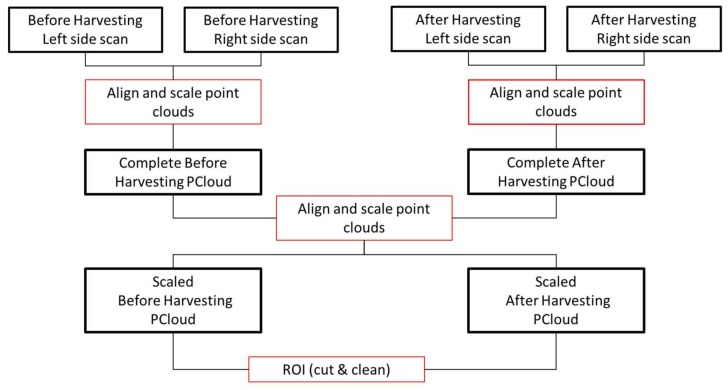
The workflow scheme for point cloud alignment and filtering for each LiDAR orientation.

The alignment and filtering of the point clouds were performed using CloudCompare software (CloudCompare 2.9.1 GNU License, Paris, France) in the following sequence (Figure 3): (1) The scanned row was delimited in the raw data using physical references and a filter distance, so that the maximum values of the Y and Z point coordinates (while the X coordinate corresponded to the advance of the tractor) considered to belong to that row were limited in the point cloud representation software; (2) The point clouds generated by LiDAR 1 (facing sideways) and LiDAR 2 (facing upwards) corresponding to the scans on the left and right sides were aligned and scaled manually. In this manner, both point clouds (left and right) were merged as follows: In the CloudCompare software a manual selection of known and immutable points belonging to the fixed references (pre and post-harvesting) was performed, and both point clouds were aligned using its alignment tool, based on calculating the transformation rigid transformation matrix. An RMS error of less than 3 cm (average RMS error = 2.51 cm) was maintained in all alignments; (3) Pre- and post-harvesting point clouds were generated and aligned in the same manner; (4) The point clouds were simplified and cleaned by removing the data corresponding to the ground and ruling out points for being out of the study area. For this purpose, fixed box-shaped references were used as follows: a rectangular region of interest (ROI) was defined, delimiting as study elements the points between the upper inner corners of the boxes (30 cm height above the ground) and their vertical projections.

##### Tree Row Volume Calculation Used to Estimate Biomass Loss Due to Harvesting

With the resulting point clouds corresponding to the ROI, the canopy volume was calculated. A self-developed program in R using the *alphashape3d* and *alphahull* libraries was used to compute the volume of the entire transect automatically. With this program, convex hull and alpha shape surface reconstruction algorithms were used to produce the surface enveloping the outer points of the point clouds as described in [34]. As explained by these authors, while the convex-hull algorithm does not allow for the creation of concave regions on the mesh, the minimum value of the parameter α for volume calculations using the alpha shape algorithm was determined to minimise the alpha shape function, thereby generating a closed volume with a concavity that enveloped all the outer points. Therefore, the calculated volumes of the point clouds using each of the algorithms are expected to differ, with the volume calculated using the alpha shape being considerably lower because it is better adjusted to the structure of the olive tree row. Additionally, the point density in each LiDAR dataset was automatically calculated with both algorithms for comparison before and after harvesting.

#### 2.5.3. USB Accelerometer Data Logger

**Figure 4:**
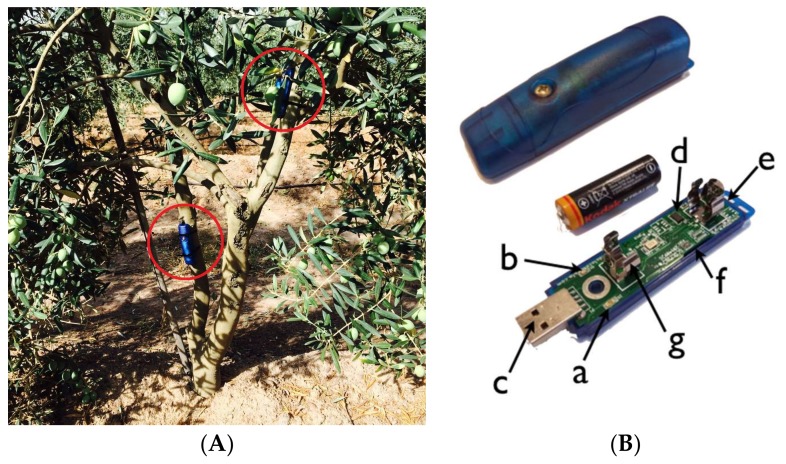
Three-axis accelerometers mounted on an olive tree (**A**) and the X16-1D accelerometer (**B**): a: the red LED data indicator, b: the blue LED status indicator, c: a type-A USB connector, d: an ADXL 345 sensor, e: an on/off button, f: a microSD card (under the circuit board) and g: the AA battery holder.

Acceleration was measured using four compact self-recording accelerometer data loggers (USB Accelerometer X16-1D, Gulf Coast Data Concepts, Waveland, MS, USA) that were set to record the acceleration (maximum of 16 g) in 3 separate axes at 100 Hz. The accelerometers present a 16-bit resolution, or 65,536 discrete counts, covering the full range of ±16 g. In this way, 2048 counts/g were obtained. The accelerometers were battery powered (type AA), and their weights and dimensions were 55 g and 26 × 26 × 104 mm, respectively. The data from the digital accelerometers were time stamped using a real-time clock (RTC) and then stored on a removable microSD card (8 GB) in simple text format. Two USB accelerometers were strapped to each tree, split between two primary branches at different heights from the ground (Figure 4).

The accelerometers were placed in different positions and branches on the same tree, to ensure that the axes of the accelerations were varied and lateral and that forward movements did not correspond to the same coordinates of the space between the accelerometers. For the analysis of these forces and accelerations suffered by the olive tree during harvesting, proprietary software was developed and used in the statistical R software. For the sake of simplicity, the *x*/*y*/*z* axes data were combined into a single vector sum (RMS) prior to their analysis. This process combined all directions into one summed value to ensure that the values could be compared between different trees without the need for equal orientations of the accelerometers.

## 3. Results and Discussion

### 3.1. Harvesting Efficiency

The fruit yield was approximately 6300 kg·ha^−1^ for ‘Manzanilla de Sevilla’ and 17,100 kg·ha^−1^ for ‘Manzanilla Cacereña’. The average time needed to harvest a hectare was less than two hours, regardless of variety (Table 3). As expected, this time decreased significantly when the harvester advanced at the 3 km/h nominal speed compared to when it advanced at 2 km/h. The high efficiency of mechanical harvesting was also demonstrated by the fruit removal values, which were equal to or higher than 97%. The lowest values were found with the machine advancing at 2 km/h and 430 Hz, although the differences among the treatments were not significant. However, note that as mentioned in Section 2.1, fruit removal decreases considerably at beating frequencies below 430 Hz. Furthermore, most of the removed fruits were intercepted by the harvester and were received in conveyor belt transport system buckets because the percentages of fruits that had fallen on the ground were lower than 3.5%. For both varieties, the highest fruit losses to the ground were found when the harvester was working at 2 km/h and 430 Hz, although the differences between the harvesting treatments were significant only for ‘Manzanilla Cacereña’. Similar time to harvest and fruit removal values have been reported at a nominal speed of 3.5 km/h and 480 Hz beating frequency in the same orchard [5]. Even though the early stage of maturity at which they were harvested was associated with a high fruit retention force, the high removal of fruits was likely due to their weight, which was greater than 3 g each [5,35].

### 3.2. Olive Fruit Damage Caused by Mechanical Harvesting

The fruit weight was inversely related to the fruit production. The average values were 4.8 g for ‘Manzanilla de Sevilla’ fruits and 4.1 g for ‘Manzanilla Cacereña’, regardless of the harvesting treatments. The fruits of ‘Manzanilla Cacereña’ showed a greater tolerance to damage by bruising and cutting than the ‘Manzanilla de Sevilla’ fruits (Table 4), as previously reported for both varieties cultivated under SHD conditions [5]. The greatest tolerance to bruising of the ‘Manzanilla Cacereña’ fruit was related to the greater thickness of the cuticle and the area of the cuticle per epidermal cell [9].

None of the parameters related to fruit damage were modified by the harvesting conditions in the ‘Manzanilla Cacereña’ fruits. The proportion of cut fruits decreased with the nominal speed and with the beating frequency; however, these differences were not significant. For ‘Manzanilla de Sevilla’ fruits, the harvesting treatments did not affect the incidence of bruising either. Nevertheless, significant differences were found between the treatments in terms of the cut damage, firmness, and colour of the fruits. The advance at the nominal speed of 3 km/h, compared to 2 km/h, led to a loss of firmness and luminosity in the detached fruits that showed more brownish tones. Therefore, the mean values of the CI also decreased. However, the primary differences were observed in the proportion of cut fruits. The lowest percentage was found when the harvester advanced at 2 km/h, regardless of the beating frequency. For these conditions, approximately 9.5% of the fruits were affected. When the nominal speed increased to 3 km/h, this damage also increased to 17%, a value similar to that reported in hedgerows harvested at 3.5 km/h nominal speed and 480 Hz beating frequency [5]. Similar results were also obtained in the previous year, even though the decrease in the beating frequency from 470 to 430 Hz with the harvester advancing at a 2 km/h nominal speed led to a reduced of the proportion of cut fruits that was significant in the case of ‘Manzanilla de Sevilla’ (data not shown).

### 3.3. Structural Olive Tree Damage Caused by Mechanical Harvesting

#### 3.3.1. LiDAR Results

In this study, a total of 90 olive trees that were laid out in hedges were scanned for two harvesting treatments per variety. Regarding the biomass losses estimate, Table 5 summarises the results of the field tests, implementing the point cloud generation and volume analysis of olive trees (the methodology was previously explained) both before and after the harvest. Two scan repetitions for each transect of the plot were made with each LiDAR sensor; thus, the mean data of the two repetitions are presented in Table 5.

As shown in Table 5, a lower point density has been recorded in all cases in the point clouds generated after harvest, corresponding to a lower amount of biomass on which to bounce the LiDAR sensor light beam, which resulted in a smaller surface enclosing the point clouds.

The average biomass volume variation in the harvest was 1.11 m^3^, and it was fairly uniform in both varieties, except for that in the Manzanilla de Sevilla variety with 2 km/h and 470 Hz treatment, for which it was considerably lower.

In most cases, the upper LiDAR obtained a slightly smaller tree volume than the lateral one. This finding could be explained because due to its position and orientation, some points that remain on the interior of the canopy can be occluded from its laser beam.

The minimum alpha value calculated in the alpha-shape function for each plot was (in most cases) close to 3, with a minimum of 3.05 and a maximum of 3.50. Due to the complexities of these surfaces, the junction points must be further apart to form a triangular mesh, which leads to higher alpha values calculations. Unlike the work performed by [34], in which a single isolated tree was analysed and modelled and an alpha of 1 or even 0.75 was obtained, the value close to 3 is justified because a more complex mesh must be created to enclose an entire tree transect.

The accuracy of the data presented in Table 6, where no ground control points have been taken, is determined by the accuracy of the point cloud alignment described in Section 2.5.2.2 and the angular resolution of the sensor presented in Section 2.5.1.

The value of the volume obtained by the convex hull function was not representative because it included too large a volume (as expected), coarsely joining the edge points of the point cloud.

**Figure 5:**
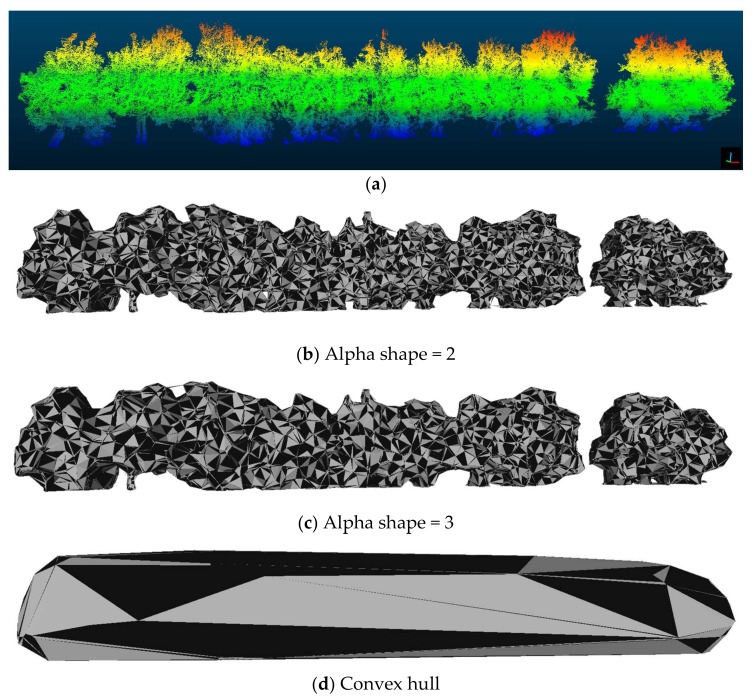
(**a**) A tree-row point cloud representation coloured by height; (**b**) a tree-row surface reconstruction using the α-shape algorithm with an α value = 2; (**c**) a tree-row surface reconstruction using theα-shape algorithm with an α value = 3; and (**d**) a tree-row surface reconstruction using the convex hull algorithm.

As shown in Figure 5, some of the plots missed some trees. In these cases, the volume of the tree wall was calculated separately, grouping the two large sets of trees together (as in the left part with eight trees and the right part with two isolated trees). This process avoided the calculation of the space that would “occupy” the missing tree, and it generates a fictitious value.

#### 3.3.2. Accelerometer Results

**Figure 6:**
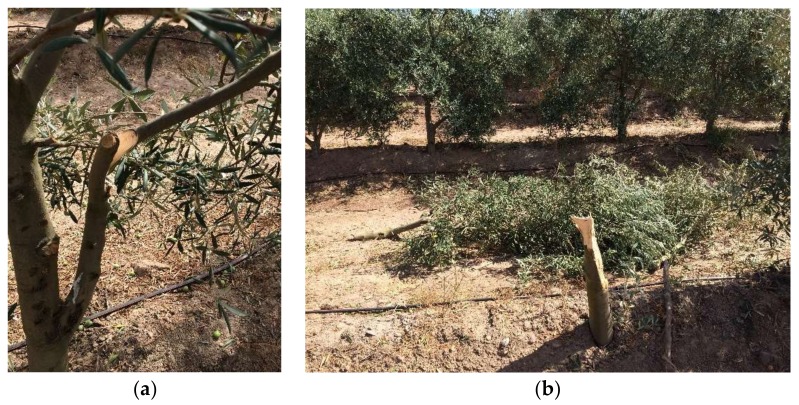
Olive tree with branch damage (**a**) and a tree tear (**b**) caused by the harvester in the same commercial field where the trials were conducted.

A total of 36 olive trees were monitored using USB accelerometers to measure and record the vibrations in the trees in both experimental trials. Table 6 shows the maximum root mean square acceleration (RMS) and the vibration time of the tree recorded by the accelerometer. An increase in the beating frequency (from 430 to 470 Hz) caused an increase in the maximum acceleration of 40% in the ‘Manzanilla Cacereña’ variety and 70% in the ‘Manzanilla de Sevilla’ variety. A change in the nominal speed of the harvester did not result in any significant differences in the maximum acceleration, however; but it reduced the vibration time per tree by 20%. At the maximum frequency, it was possible to break the branches or even to cut the olive trees (Figure 6). This plant damage may be an indirect cause of concealed losses [36,37].

## 4. Conclusions

The efficiency of fruit removal by over-the-row harvesters in SHD olive orchards is high, reaching approximately 100%, with most fruit intercepted by the machine moving at approximately two hours per hectare. However, the nominal speed and beating frequency can negatively influence the fruit and/or increase canopy damage, particularly for the least tolerant varieties. Advancing at a controlled nominal speed is useful for minimising the proportion of fruits with cuts, at least for the harvesters currently available on the market. The beating frequency of the bow-shaped poles is key to obtaining a high fruit removal efficiency, and it should also be controlled to decrease the damage to the fruit, primarily by cuts.

According to the results obtained by using the LiDAR sensors to estimate the volume and biomass losses during harvesting, the following conclusions can be drawn:

- The evaluated methodology for on-board data collection and off-line volume measuring was able to determine the row-tree volume properly with a high level of localization. Even when using a low-cost odometry reference system, the correct spatial localization of laser scans was achieved, allowing for the generation of accurate point clouds in the tree rows.
- Field experiments using the LiDAR sensors indicate that in all cases, there is a notable loss of biomass between the pre- and post-harvest mass. This biomass combines both the fruit that is harvested and the structure of the tree itself. For this reason, the most relevant indicator of tree damage was established via the study of the canopy volume through the alpha function for surface generation. In this manner, information was obtained from the outer points of the cloud, producing a surface that envelopes the object and shapes the geometry of the tree.
- The average biomass volume variation in the harvest was 1.11 m^3^, and it was fairly uniform in both varieties.
- Two different laser scan orientations (one scanning upwards and the other one scanning sideways) were tested with the same scanning range to compare the point cloud densities and accuracies in the volume measurements. The results show that the upwards LiDAR obtained a slightly smaller tree volume than the lateral one, which could be explained because of its position and orientation; the points remaining on the inside-bottom part of the canopy were occluded from its laser beam.
- The agricultural use of on-board sensing techniques, such as LiDAR sensors, could reduce costs in the near future, in terms of yield estimation, tree damage calculation or help with autonomous navigation. As mentioned in previous studies referred to in the introduction (for example, in [20,24,29]) the complexity in the use of LiDAR sensors and the analysis of the large amount of data generated by these systems still causes them to have long processing times, while specific software must be developed to obtain accurate user-friendly information. Thus, the authors can also conclude that these systems still lack the robustness and quicker response needed to be pre-commercially deployable under unstructured scenarios such as real field conditions.

## Figures and Tables

**Table 1:** LMS111 technical data.

Operational range	0.5 to 20 m
Scanning field of view	270°
Scanning Frequency	50 Hz
Angular resolution	0.5°
Light source	Infrared (905 nm)
Enclosure rating	IP 67

**Table 2:** Transformation and translation angles applied to the LiDAR data.

-	LiDAR 1 (Facing Sideways)		LiDAR 2 (Facing Upwards)	
Side of the scan	Left	Right	Left	Right
Roll φ	0	0	Pi/2	Pi/2
Pitch θ	Pi/2	−Pi/2	0	0
Yaw ψ	0	0	Pi/2	−Pi/2

**Table 3:** Harvesting efficiency for each nominal travel speed and beating frequency.

-	‘Manzanilla Cacereña’			‘Manzanilla de Sevilla’		
	3 km/h-470 Hz	2 km/h-470 Hz	2 km/h-430 Hz	3 km/h-470 Hz	2 km/h-470 Hz	2 km/h-430 Hz
Time to harvest (h·ha^−1^)	1.1a	1.6b	1.8b	1.1a	1.7b	1.5b
Fruit removal (%)	99.5	99.7	96.8	99.9	99.5	98.8
Fruit on ground (%)	2.1ab	1.4a	2.3b	1.9	2.0	3.3

Lowercase letters indicate significant differences in treatments for each cultivar at *p* < 0.05.

**Table 4:** Fruit damage for each nominal travel speed and beating frequency.

Fruit Characteristics	‘Manzanilla Cacereña’			‘Manzanilla de Sevilla’		
	3 km/h-470 Hz	2 km/h-470 Hz	2 km/h-430 Hz	3 km/h-470 Hz	2 km/h-470 Hz	2 km/h-430 Hz
Bruising Incidence	1.3 A	1.3 A	1.3 A	1.5 B	1.6 B	1.6 B
Cut fruit (%)	9.0 A	7.3 A	1.7 A	16.7 bA	9.3 aA	9.7 aB
Firmness (N·cm^−2^)	44.5 A	44.5 A	45.0 A	46.0 aB	47.0 abB	47.5 bB
Colour Index (CI)	23.8 A	23.8 A	24.8 A	23.2 aA	25.1 bB	24.6 bA

Lower case letters in the same row indicate significant differences among the treatments for each cultivar at *p* < 0.05; Upper case letters in the same row indicate significant differences between the cultivars for each treatment at *p* < 0.05.

**Table 5:** Tree row volume scanned with LiDAR sensors.

		Scan (Before Harvest, BH; After Harvest AH)	Average Volume (Convex Hull)	Average Volume (Alphashape)	∆Volume (V_BH_–V_AF_) Using Alphashape	Average α Value	Average Point Cloud Density	∆Point Cloud Density
**‘Manzanilla Cacereña’**								
**3 km/h (470 Hz)**	LiDAR 1 lateral position	BH	51.32 m^3^	38.63 m^3^	-	3.20	941,169	-
		AH	49.94 m^3^	37.60 m^3^	1.02 m^3^	3.15	821,556	119,613
	LiDAR 2 upper position	BH	49.24 m^3^	40.93 m^3^	-	3.30	1,073,330	-
		AH	45.78 m^3^	39.05 m^3^	1.87 m^3^	3.30	812,332	260,998
**2 km/h (470 Hz)**	LiDAR 1 lateral position	BH	66.58 m^3^	58.01 m^3^	-	3.00	1,307,456	-
		AH	62.68 m^3^	56.96 m^3^	1.05 m^3^	3.10	1,156,895	150,561
	LiDAR 2 upper position	BH	61.17 m^3^	56.83 m^3^	-	3.10	984,536	-
		AH	57.22 m^3^	55.42 m^3^	1.41 m^3^	3.05	843,732	140,804
**‘Manzanilla de Sevilla’**								
**3 km/h (470 Hz)**	LiDAR 1 lateral position	BH	57.52 m^3^	48.43 m^3^	-	3.20	969,025	-
		AH	56.01 m^3^	46.78 m^3^	1.64 m^3^	3.10	738,178	230,847
	LiDAR 2 upper position	BH	55.79 m^3^	48.19 m^3^	-	3.00	1,048,921	-
		AH	55.78 m^3^	47.08 m^3^	1.10 m^3^	3.00	875,640	173,281
**2 km/h (470 Hz)**	LiDAR 1 lateral position	BH	67.16 m^3^	47.98 m^3^	-	3.50	1,090,924	-
		AH	67.05 m^3^	47.39 m^3^	0.59 m^3^	3.50	984,563	106,361
	LiDAR 2 upper position	BH	67.61 m^3^	45.09 m^3^	-	3.50	1,142,788	-
		AH	69.42 m^3^	44.87 m^3^	0.21 m^3^	3.50	1,089,874	52,914

**Table 6:** Measurements of the maximum acceleration and vibration time suffered by olive trees using USB accelerometers.

	**‘Manzanilla Cacereña’**					
	**Tree 1**		**Tree 2**		**Total**	
**Treatments**	**Max. Ac (g)**	**T (s)**	**Max. Ac (g)**	**T (s)**	**Mean Max. Ac (g)**	**Mean T (s)**
3 km/h-470 Hz	7.07	3.25	6.62	3.13	6.85	3.19
2 km/h-470 Hz	7.35	4.38	7.07	5.25	7.21	4.81
2 km/h-430 Hz	5.46	4.12	4.71	4.00	5.08	4.06
	**‘Manzanilla de Sevilla’**					
	**Tree 1**		**Tree 2**		**Total**	
**Treatments**	**Max. Ac (g)**	**T (s)**	**Max. Ac (g)**	**T (s)**	**Mean Max. Ac (g)**	**Mean T (s)**
3 km/h-470 Hz	7.04	4.50	5.14	3.50	6.09	4.00
2 km/h-470 Hz	7.40	4.68	8.24	5.37	7.82	5.25
2 km/h-430 Hz	4.25	4.31	4.83	4.75	4.54	4.53
